# Pathology of the Calcified Zone of Articular Cartilage in Post-Traumatic Osteoarthritis in Rat Knees

**DOI:** 10.1371/journal.pone.0120949

**Published:** 2015-03-25

**Authors:** Melissa Schultz, Jeremy Molligan, Lew Schon, Zijun Zhang

**Affiliations:** 1 Center of Anatomical Science, Saint Louis University, St. Louis, Missouri, United States of America; 2 Orthobiologic Laboratory, Medstar Union Memorial Hospital, Baltimore, Maryland, United States of America; University of Pittsburgh, UNITED STATES

## Abstract

**Objectives:**

This study aimed to investigate the pathology occurring at the calcified zone of articular cartilage (CZC) in the joints afflicted with post-traumatic osteoarthritis (PTOA).

**Methods:**

Rats underwent bilateral anterior cruciate ligament (ACL) transection and medial meniscectomy to induce PTOA. Sham surgery was performed on another five rats to serve as controls. The rats were euthanized after four weeks of surgery and tibial plateaus were dissected for histology. The pathology of PTOA, CZC area and the tidemark roughness at six pre-defined locations on the tibial plateaus were quantified by histomorphometry.

**Results:**

PTOA developed in the knees, generally more severe at the medial plateau than the lateral plateau, of rats in the experimental group. The CZC area was unchanged in the PTOA joints, but the topographic variations of CZC areas that presented in the control knees were reduced in the PTOA joints. The tidemark roughness decreased in areas of the medial plateau of PTOA joints and that was inversely correlated with the Mankin’s score of PTOA pathology.

**Conclusion:**

Reduced tidemark roughness and unchanged CZC area differentiate PTOA from primary osteoarthritis, which is generally believed to have the opposite pathology at CZC, and may contribute to the distinct disease progression of the two entities of arthropathy.

## Introduction

Post-traumatic osteoarthritis (PTOA) is a form of arthropathy secondary to joint injury. It is estimated that 12% of all osteoarthritic complaints in the hip, knee, and ankle are post-traumatic [1]. Clinically, PTOA has a younger patient population than primary osteoarthritis (OA). Therefore, effective treatments for primary OA, such as joint replacement, fusion and restriction, may not be applicable or acceptable for PTOA patients [2]. The clinical outcome of PTOA has been virtually unchanged for the past 50 years [3]. It is generally believed that the mechanical instability caused by joint injury is the predominant pathogenesis of PTOA [4], [5]. The PTOA pathology, particularly what distinguishes PTOA from primary OA has not been fully understood [6].

Articular cartilage is organized in a zonal structure. The calcified zone of cartilage (CZC) is the deepest zone where cartilage matrix is calcified. It is separated from the other zones of cartilage by the tidemark. The CZC borders the subchondral bone with the cement line. Matrix mineralization in CZC allows gradual transition of mechanical properties between cartilage and bone [7]. It would become a focal zone, however, during the development of PTOA whose pathology features mechanical disorders of the joint. In addition, the permeability of the CZC is five times less than that of the uncalcified cartilage [8], [9], suggesting that the CZC serves as a physiological barrier between the tissue compartments of bone and cartilage. Therefore, changes in the CZC during PTOA development have both mechanical and biological consequences.

The tidemark is also referred to as the chondro-osseus junction [10]. The shape of the tidemark is often described as having a “gentle waviness”. The degree of undulation, or roughness, of the tidemark is responsive to mechanical stress [11]. The tidemark is significantly less rough than the cement line, which joints CZC with subchondral bone [12]. This may suggest that the roughness of the tidemark and cement line is regulated by the mechanical properties of the adjacent cartilage and bone, which undergo significant changes in OA and PTOA [13], [14].

In response to joint instability in PTOA, bone and cartilage are continually remodeling [15]. In the (uncalcified) cartilage, chondrocytes undergo apoptosis and matrix degrades [16]. Simultaneously, active calcification is triggered in the subchondral bone, resulting in the formation of a thicker subchondral bone plate [17]. In this context, changes in CZC are inevitable but few investigations focused on the CZC in PTOA.

In this study, PTOA in rat knees was induced by transaction of the anterior cruciate ligament (ACL) and medial meniscectomy. The PTOA pathology, CZC areas and tidemark roughness at tibial plateaus were quantified by histomorphometry.

## Materials and Methods

### 1. Animal model of PTOA

Ten male Wistar rats (Harlen Laboratories, Indianapolis, IN), 12 weeks of age, were used for this study (approved by Saint Louis University Institutional Animal Care and Usage Committee; Protocol 2242). The rats were individually housed and the cage numbers were used to randomly assign the animals in experimental and control groups. Rats were anesthetized by an intraperitoneal injection of a cocktail of ketamine and zylazine and both knees were prepared for surgery. A parapatellar incision was made to expose the knee joint. The ACL was transected and the medial meniscus was resected. The joint capsule and skin were closed separately with 6.0 vicryl suture. Sham surgery was performed on the rat knees in the control group and consisted of opening the joint capsule but no ACL transection and meniscectomy. The operated limbs were not immobilized and rats were allowed to access food and water *ad lib*.

All animals survived and had no limitation of mobility. Rats were euthanized four weeks post-operatively. A total of 19 knees (one in the experimental group was eliminated due to infection) were dissected and fixed in 4% paraformaldehyde. Specimens were decalcified using Acumate Rapid Decalcifying Solution (Sigma-Aldrich Co., St. Louis, MO). The knees were then disarticulated and the tibial portions were coronally bisected, using a custom made jig for accurate positioning and reproducible sections. The tibial parts of the knees were embedded in TissueTek OCT (Sakura, Torrance, CA) after being balanced in 15% and 30% sucrose solutions. Tissue sections (6 μ) were cut using a cryostat and stained with Safranin-O, Fast Green, and Hematoxylin. Images were taken with a Leica DMI 4000 light microscope and analyzed with ImageJ (NIH).

### 2. Image analyses

**Fig 1:**
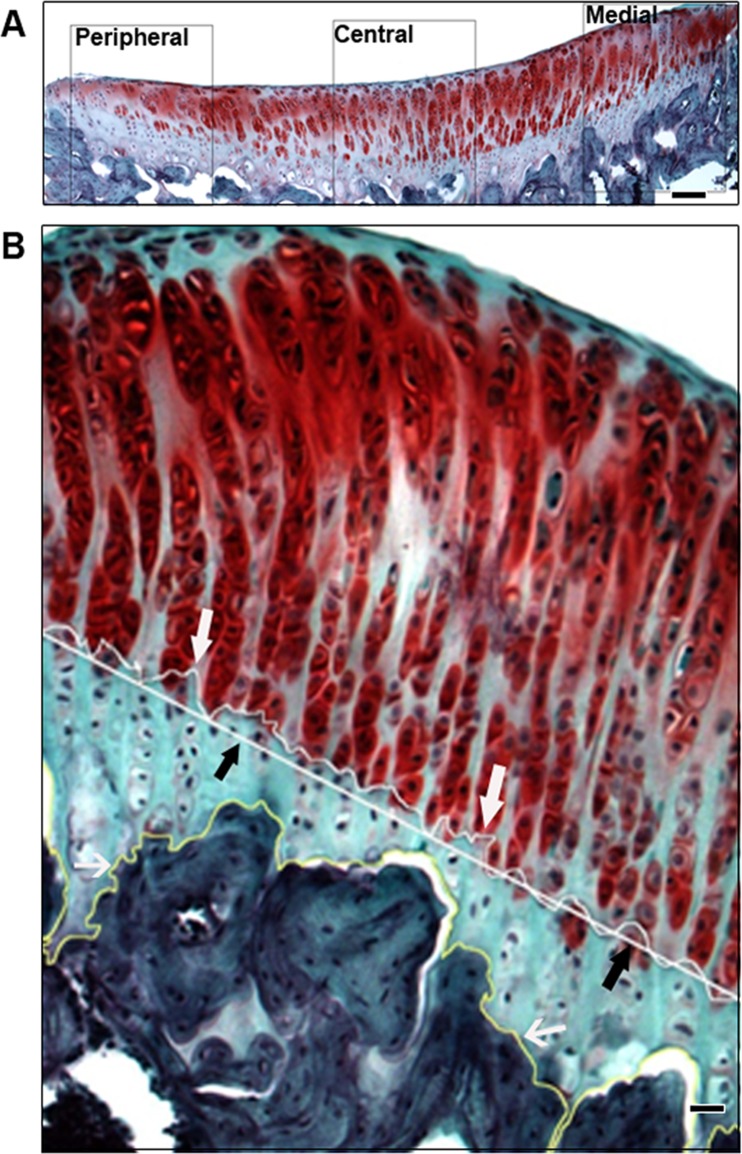
A) Diagram of selected locations on tibial plateau for Mankin’s score and CZC measurements. Showing here is a lateral tibial plateau, where standardized areas are defined in the evenly divided three portions of the plateau: peripheral portion, near the edge of the plateau; the central portion, in the middle of the plateau; and the medial portion, close to the cruciate ligaments. Three areas are defined in the same way on the medial plateau (bar = 50μm). B) CZC measurements, using ImageJ. The tidemark was traced for true length (L, white arrows). The straight line (black arrows) was drawn, as L0, for calculation of tidemark roughness. The cement line between CZC and subchondral bone was also traced (open arrows) for measurements of CZC area between the tidemark and cement line (bar = 20μm).

For histological analysis, each medial and lateral tibial plateau was divided into three areas (Fig. 1A): medial (closest to the tibial spine), central (center of the tibial fossa), and peripheral (closest to the edges of tibial plateau). An equally sized, consistently located portion from each third of the medial or lateral tibial plateau was selected for histomorphometry. From the same specimen, the same locations on two or more consecutive sections were analyzed and averaged in order to reduce systematic errors.

On each selected imaging area, the degree of PTOA, CZC area and the tidemark roughness were quantified. The Mankin’s scoring system was used to assess the severity of cartilage degeneration in cellularity, matrix biochemistry and surface integrity [18]. Specimens were graded on a scale of zero, representing healthy cartilage, to eleven representing severe OA.

CZC area was measured by tracing the borders of the CZC using the freehand selection tool of ImageJ. Tidemark roughness (R) was calculated using the following equation: R = L/L0, where L is the true length of the tidemark and L0 is the anticipated projection length [12]. The true length of the tidemark (L) was traced using the freehand line tool and anticipated projection length (L0) was measured using the straight line tool (Fig. 1B).

#### Statistical analyses

Data are expressed as mean ± standard deviation. T tests were used to assess differences between PTOA and control knees at corresponding locations in Mankin’s score, CZC area and tidemark roughness. One way ANOVA, followed by *post hoc* Tukey’s test, was used to analyze the Mankin’s score, CZC area, and tidemark roughness among the six locations on both medial and lateral plateaus in the same study group. Pearson’s Correlation was used to determine the relationship between the Mankin’s scores and CZC areas or tidemark roughness in the PTOA knees. The significance level was set at p < 0.05.

## Results

### 1. Cartilage degeneration in PTOA joints

**Fig 2:**
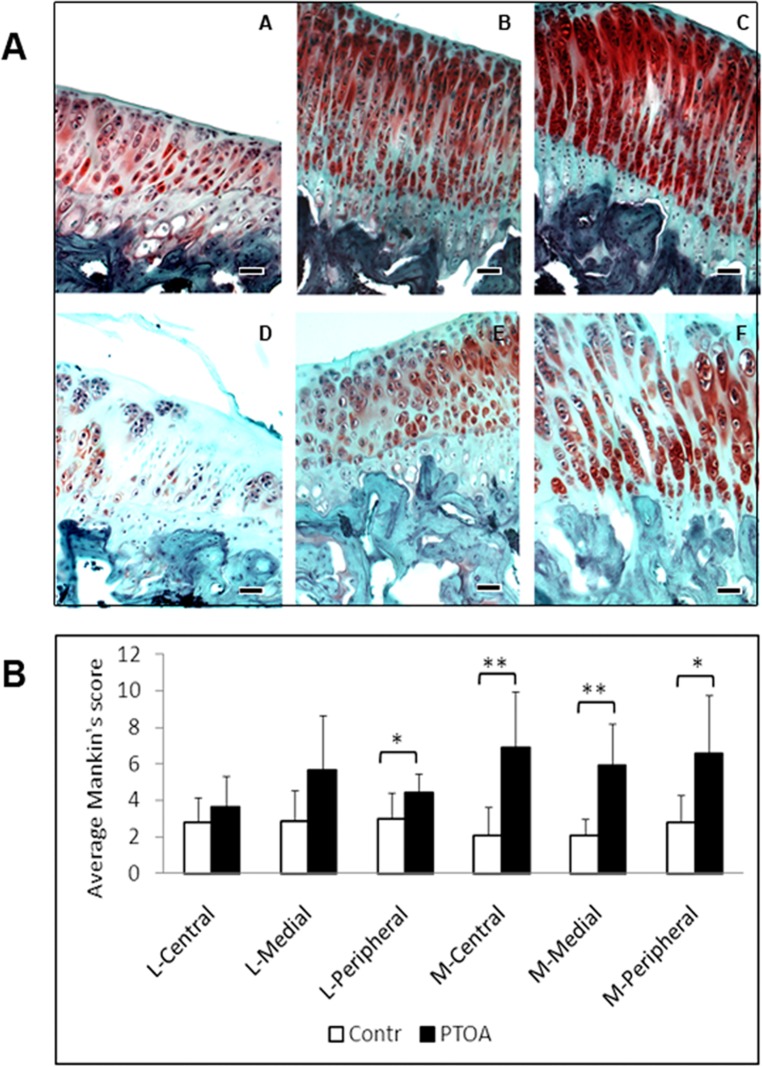
A) Histology of PTOA knees. A, B and C are the peripheral, central and medial regions of the medial plateau in the control knees. D, E, and F are the peripheral, central and medial regions of the medial plateau of the PTOA knees, respectively. While the cartilage in the control knees is intact and uniformly stained for extracellular matrix, the cartilage in the PTOA joints shows clustering cells, decreased and uneven staining of proteoglycans, and surface fibrillation (Safranin-O/Fast green/Hematoxylin staining). B) Mankin’s scores of six selected locations on the tibial plateau of the PTOA and control knees. PTOA developed in all three locations on the medial plateau and the peripheral area of the lateral plateau after four weeks of ACL transection and meniscectomy. Note: * indicates p < 0.05; ** p < 0.001. L = lateral plateau; M = medial plateau; bar = 20μm.

The pathology of PTOA induced by meniscectomy and transaction of ACL included erosion of cartilage surface, loss of proteoglycan from the matrix, formation of chondrocyte clusters (Fig. 2A). The Mankin’s score in the experimental group showed significant cartilage degeneration at all three selected locations of the medial plateau and the peripheral portion of the lateral plateau, compared with the corresponding locations in the control group. The Mankin’s scores for the central and medial areas of the lateral plateau in the PTOA knees were not statistically different from the control (Fig. 2B). As measured by the Mankin’s score, the central area of medial plateau had the most severe PTOA among the involved areas on the tibial plateau. There was, however, no statistical difference among the Mankin’s scores of the six assigned areas on the plateaus of PTOA knees (p = 0.07).

### 2. PTOA associated CZC pathology

**Fig 3:**
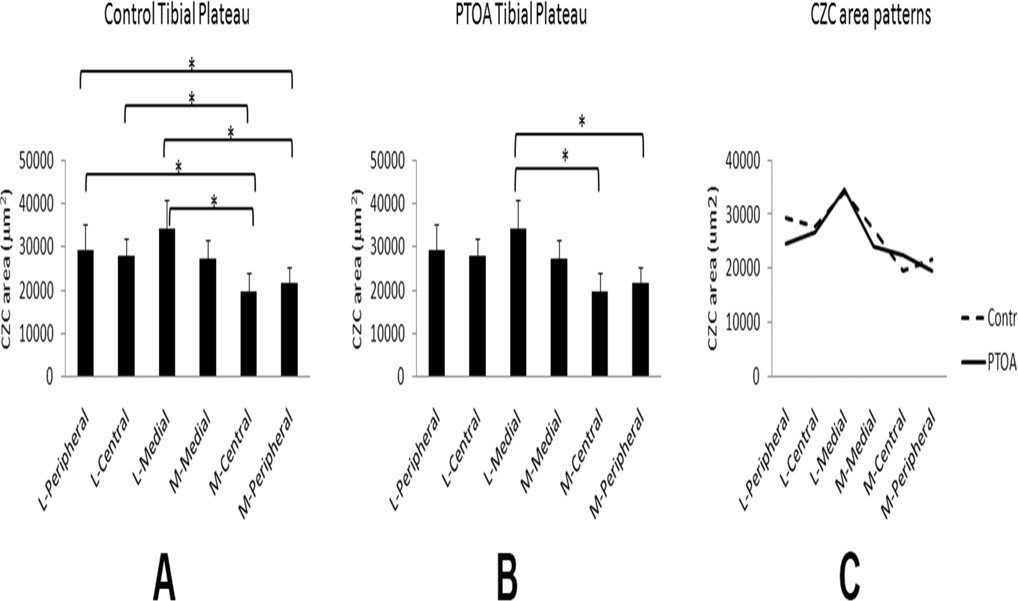
Measurements of the CZC area in six selected locations on the tibial plateau of the PTOA and control knees. A: The topographic distribution of CZC area is uneven across the tibial plateau in the control knees. The CZC areas at the peripheral and central regions on the lateral plateaus are larger than the corresponding locations on the medial plateau. In addition, there are significant differences in CZC area among several asymmetric locations between the lateral and medial plateaus. B: On the tibial plateaus of the PTOA joints, the number of significant differences in CZC area among different locations is reduced, as compared with the control tibial plateaus. C: Although the distribution pattern of CZC area in the PTOA joints is altered, the CZC areas sampled at each location of the PTOA tibial plateaus are unchanged when statistically compared with the corresponding locations in the control knees. Note: * indicates p < 0.05. L = lateral plateau; M = medial plateau.

The CZC area varied significantly among the six defined locations across the tibial plateau. Within the control group, the CZC area at the central area of the medial plateau was the smallest and the medial area of the lateral plateau was the largest. Among several locations, the CZC areas were statistically different, such as medial versus lateral plateau at the central and peripheral areas. The unevenness of CZC areas was also demonstrated with differences among asymmetric locations between the lateral and medial plateaus, such as the peripheral area on the lateral plateau vs. the central area on the medial plateau (Fig. 3A). A significant change in the PTOA knees was the disappearance of many statistical differences in the CZC area between different locations across the tibial plateau. The remaining differences between CZC areas in the PTOA knees were the medial area of the lateral plateau versus the peripheral and central areas of the medial plateau. In the PTOA knees, the smallest CZC area was in the peripheral area of the medial plateau, whereas the largest was, the same as the control, at the medial area of the lateral plateau (Fig. 3B). Each CZC area of the six locations on the tibial plateau in the PTOA joints did not differ significantly from the corresponding locations in the control group, although four of the six areas on the tibial plateau in the PTOA joints showed a tendency of reduced CZC area (Fig. 3C).

**Fig 4:**
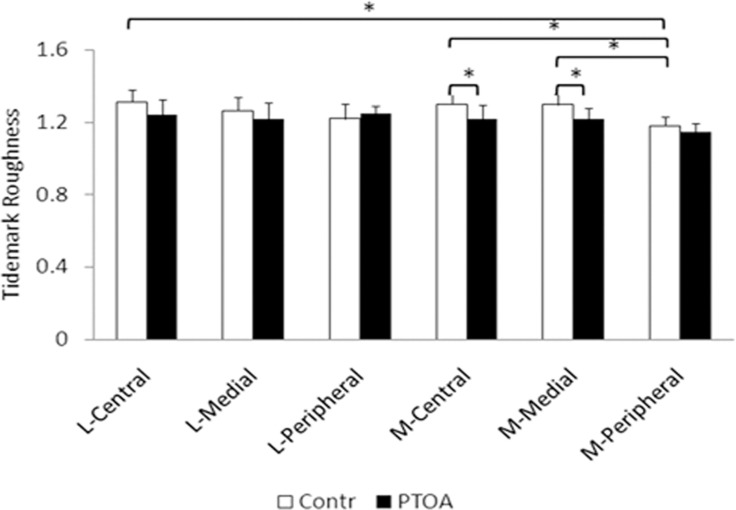
The tidemark roughness at the six selected locations on the tibial plateau of the PTOA and control knees. The tidemark roughness in the control knees varies from area to area on the tibial plateau. In the PTOA knees, there are not regional differences in tidemark roughness. In addition, the generally reduced tidemark roughness in the PTOA knees is statistically significant at the central and medial areas of medial plateau, when compared with the controls. Note: * indicates p < 0.05. L = lateral plateau; M = medial plateau.

The tidemark roughness was not equal across the tibial plateau in the control group. The lowest tidemark roughness was at the peripheral area of the medial plateau. There were significant differences in the tidemark roughness between several pairs of locations, such as the peripheral area versus medial and central areas on the medial plateau and the central area of the lateral plateau. The differences of tidemark roughness among the six defined locations on the tibial plateau, however, did not exist in the PTOA knees. Compared with the control, tidemark roughness in the PTOA knees was reduced in general, except at the peripheral area of the lateral plateau. When the tidemark roughness was compared between the PTOA and control knees at the corresponding locations, it was found that tidemark roughness was significantly reduced in the PTOA joints at the central and medial areas of the medial plateau (Fig. 4).

### 3. Correlation among PTOA pathology, CZC area and tidemark roughness

**Fig 5:**
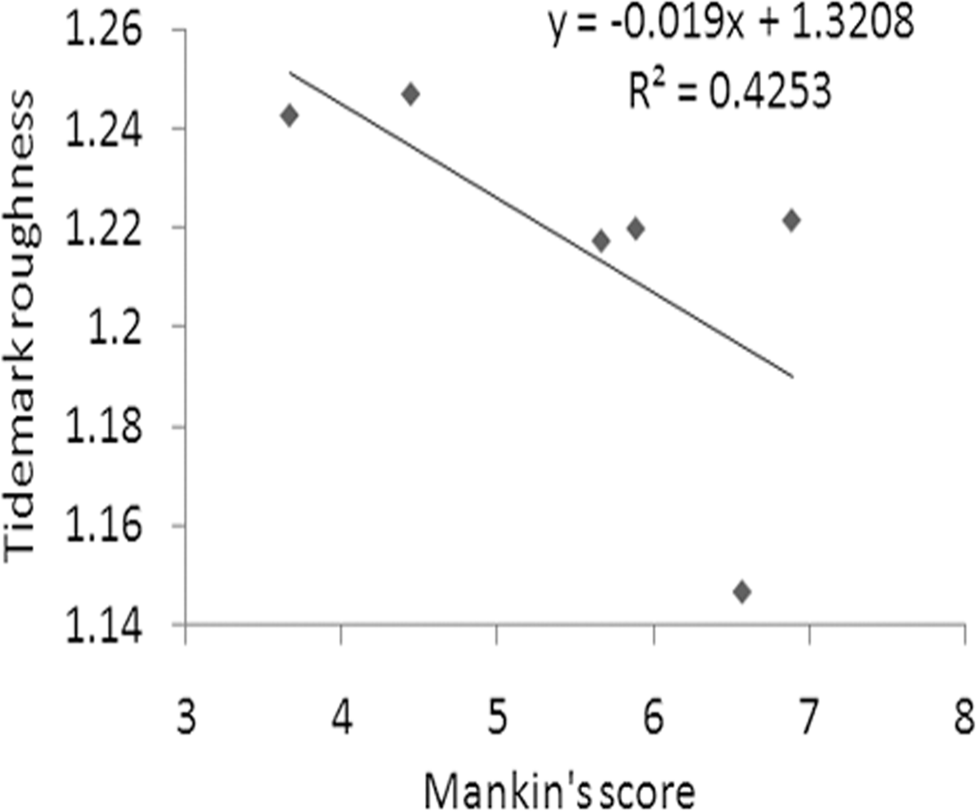
Correlation between Mankin’s score and tidemark roughness. In the PTOA knees, Mankin’s score and tidemark roughness are reversely correlated (p < 0.05).

No correlation was found between the Mankin’s scores and CZC areas. The Mankin’s scores and tidemark roughness in the PTOA joints were inversely correlated (r = -0.65; p = 0.02; Fig. 5). The CZC area and tidemark roughness in the PTOA joints did not correlate (p = 0.44).

## Discussion

The integrity of the ACL and meniscus is essential for the normal kinematics of the knee joint. Surgical damage to the ACL and meniscus applied in this study significantly destabilized the joints. When the abnormal stress developed in the joint excesses the adaptability of cartilage, cartilage degeneration occurs [19]. In this study, PTOA developed in all three selected locations on the medial plateau and the peripheral portion of the lateral plateau, but the central and medial portions of the lateral plateau were spared from significant cartilage degeneration. The severity of PTOA was the greatest in the peripheral portion of the medial plateau, followed by the central portion of the medial plateau. The intraarticular pathology developed on the medial plateau is in line with the surgical removal of medial meniscus.

The pathologies of cartilage and subchondral bone in PTOA have been subjects of extensive investigations [20], [21]. The CZC is a thin layer of cartilage (about 1/25 of the uncalcified cartilage on human femoral condyles [22]). It is critically located to unite articular cartilage and subchondral bone and, thus, is in a position that could well influence the progression of PTOA. At the calcification front of the CZC, the tidemark has been found to be metabolically active in primary OA [23]. In the mandibular condyle, though it is fibrocartilage, tidemark appears wavy in load-bearing areas and relatively flat and smooth in non-load bearing areas [24], indicating the shape of tidemark is regulated to some extent by mechanical loading of the joint. Since biomechanical instability dominates PTOA pathology, the CZC pathology could be significant to the development of PTOA.

This study employed a model of acute joint injury leading to accelerated joint degeneration. Compared with the controls, the CZC area in the PTOA joints remained constant or decreased in most of the locations on the tibial plateau. In a normal joint, the CZC maintains a constant thickness throughout life, because of the equilibrium of calcification advancing into uncalcified cartilage and absorption by endochondral ossification at the opposite end [25]. This balance relies on physiological cellular activities and a normal biomechanical environment [26]. In the PTOA joints, mechanical instability and histological pathology were evident and that indeed impacted on the properties of the CZC.

It is known that CZC thickness (area) varies topographically in the same joint [27], [28]. In the control rat knees, the CZC areas in the six selected locations on the tibial plateau were different at six paired locations. This topographical variation in the CZC area on the same tibial plateau, however, was minimized in the PTOA joints—-only 2 paired locations were statistically different. It is most likely that the CZC area pattern was adjusted to respond to the biomechanical instability in the PTOA joints.

Changes of CZC area are seen in other physiological/pathological conditions. The CZC area increases as aging progresses [29–31]. In primary OA, the expansion/advancement of CZC is regarded as a primary pathology, since it may contribute to the thinning of articular cartilage [27], [32–34]. Interestingly, when OA was induced in rats by excessive running, the CZC was expanded [35]. It is noteworthy that, during aging, and in primary OA and the excessive running OA model, the joints experience accumulated loading over a long period of time, whereas the PTOA joints in the current study were destabilized in an acute fashion and joint degeneration occurred merely in four weeks. While mechanical instability is significant in PTOA, it is much subtle and developed gradually in primary OA [2]. This may also contribute to the different patterns of CZC area found in this study and in primary OA.

The calcification front—tidemark is a mechanism to equilibrate cartilage mineralization at the bone and cartilage junction. In response to a pathological environment of primary OA, tidemark is observed in duplication or multiple lines [7], [10], [30], [36]. Tidemark duplications can be directly accompanied with fibrillation on the surface of articular cartilage in OA [23], [37]. While tidemark duplication was occasionally observed in the PTOA joints in this study, the advancement of the calcification front and CZC into the uncalcified cartilage seemed not occur.

In a classic study, patients with primary OA were given two episodes (ten days apart) of tetracycline prior to the surgery of total hip replacement [23]. The tetracycline-labeled tidemark in the articular cartilage of femoral head advanced toward uncalcified cartilage. The distance between the two tetracycline labeling lines in cartilage was 2.8 times of that in bone. The results indicate that the tidemark in OA is metabolically active, leading to the expectation of a rougher tidemark. Topographically, the tidemark roughness varied between a few paired locations on the tibial plateau in the control joints. In the PTOA joints, however, no differences were found between any paired locations on the tibial plateau. Overall, the tidemark roughness decreased in the PTOA joints. The tidemark roughness was significantly reduced at the central and medial areas of the medial plateau in the PTOA joints, when compared with the control joints. Importantly, the reduced tidemark roughness was inversely correlated with the severity of cartilage degeneration. In PTOA joints, both the central and medial portions of the medial plateau, where had reduced tidemark roughness, showed the largest increases of Mankin’s scores. The peripheral portion of the lateral plateau had increased tidemark roughness in the PTOA joints, but it incurred the smallest change in Mankin’s scores. Therefore, the reduced tidemark roughness could be the CZC pathology specific to PTOA.

This study created bilateral PTOA in rat knees. In the same rat, operating on one knee joint and using the opposite joint as a control may minimize inter-individual variables, but the animals may spare the operated limb and reduce joint loading. It has been noticed that the injured joints, without bearing weight, have an increased CZC area [26]. Indeed, unilateral knee PTOA induced by a procedure similar to the one used in the present study demonstrated an increased CZC thickness [29]. In this study, surgery performed bilaterally forced animals bearing similar loads on both injured joints, which quickens the progression of PTOA [38].

In summary, tidemark roughness was locally reduced in PTOA induced by ACL transection and medial meniscectomy and this was inversely correlated with Mankin’s scores. Although CZC area is increased in primary OA, the CZC area in the PTOA model used in this study was unchanged, but the topography of CZC areas across tibial plateau was modified. These CZC pathologies provide insight to the progression of PTOA and show features that differentiate PTOA from primary OA.

## References

[1] Brown TD, Johnston RC, Saltzman CL, Marsh JL, Buckwalter JA. Posttraumatic osteoarthritis: a first estimate of incidence, prevalence, and burden of disease. J Orthop Trauma. 2006;20:739–744. 17106388 10.1097/01.bot.0000246468.80635.ef

[2] Buckwalter JA, Brown TD. Joint injury, repair, and remodeling: roles in post-traumatic osteoarthritis. Clin Orthop Relat Res. 2004;423:7–16. 15232420

[3] McKinley TO, Borrelli J Jr, D'Lima DD, Furman BD, Giannoudis PV. Basic science of intra-articular fractures and posttraumatic osteoarthritis. J Orthop Trauma. 2010;24:567–570. 10.1097/BOT.0b013e3181ed298d 20736796 PMC3662545

[4] Kramer WC, Hendricks KJ, Wang J. Pathogenetic mechanisms of posttraumatic osteoarthritis: opportunities for early intervention. Int J Clin Exp Med. 2011;4:285–298. 22140600 PMC3228584

[5] Natoli RM, Athanasiou KA. Traumatic loading of articular cartilage: Mechanical and biological responses and post-injury treatment. Biorheology 2009;46:451–485. 10.3233/BIR-2009-0554 20164631

[6] Anderson DD, Chubinskaya S, Guilak F, Martin JA, Oegema TR, Olson SA, et al Post-traumatic osteoarthritis: improved understanding and opportunities for early intervention. J Orthop Res. 2011;29:802–809. 10.1002/jor.21359 21520254 PMC3082940

[7] Mansfield JC, Winlove CP. A multi-modal multiphoton investigation of microstructure in the deep zone and calcified cartilage. J Anat. 2012; 220:405–416. 10.1111/j.1469-7580.2012.01479.x 22332832 PMC3375776

[8] Pan J, Zhou X, Li W, Novotny JE, Doty SB, Wang L. In situ measurement of transport between subchondral bone and articular cartilage. J Orthop Res. 2009; 27:1347–1352. 10.1002/jor.20883 19360842 PMC2748158

[9] Arkill KP, Winlove CP. Solute transport in the deep and calcified zones of articular cartilage. Osteoarthritis Cart. 2008;16:708–714. 10.1016/j.joca.2007.10.001 18023368

[10] Lyons TJ, Stoddart RW, McClure SF, McClure J. The tidemark of the chondro-osseous junction of the normal human knee joint. J Mol Histol. 2005;36:207–215. 15900412 10.1007/s10735-005-3283-x

[11] Oegema TR Jr, Carpenter RJ, Hofmeister F, Thompson RC Jr. The interaction of the zone of calcified cartilage and subchondral bone in osteoarthritis. Microsc Res Tech. 1997;37:324–332. 9185154 10.1002/(SICI)1097-0029(19970515)37:4<324::AID-JEMT7>3.0.CO;2-K

[12] Wang F, Ying Z, Duan X, Tan H, Yang B, Guo L, et al Histomorphometric analysis of adult articular calcified cartilage zone. J Struct Biol. 2009;168:359–365. 10.1016/j.jsb.2009.08.010 19723582

[13] Silver FH, Bradica G, Tria A. Do changes in the mechanical properties of articular cartilage promote catabolic destruction of cartilage and osteoarthritis? Matrix Biol. 2004;23:467–476. 15579313 10.1016/j.matbio.2004.08.003

[14] Burr DB, Schaffler MB. The involvement of subchondral mineralized tissues in osteoarthrosis: quantitative microscopic evidence. Microsc Res Tech. 1997;15:343–357. 10.1002/(SICI)1097-0029(19970515)37:4<343::AID-JEMT9>3.0.CO;2-L 9185156

[15] Lories RJ, Luyten FP. The bone-cartilage unit in osteoarthritis. Nat Rev Rheumatol. 2011;7:43–49. 10.1038/nrrheum.2010.197 21135881

[16] Vrahas MS, Mithoefer K, Joseph D. The long-term effects of articular impaction. Clin Orthop Relat Res. 2004; 423:40–43. 15232424 10.1097/01.blo.0000133567.28491.7d

[17] Madry H, Niek van Dijk C, Mueller-Gerbl M. The basic science of the subchondral bone. Knee Surg Sports Traumatol Arthrosc. 2010;18:419–433. 10.1007/s00167-010-1054-z 20119671

[18] Mankin HJ, Dorfman H, Lippiell L, Zarins A. Biochemical and metabolic abnormalities in articular cartilage from osteoarthritic human hips. 2. Correlation of morphology with biochemical and metabolic data. J Bone Joint Surg Am. 1971;53:523–537. 5580011

[19] Andriacchi TP, Mündermann A, Smith RL, Alexander EJ, Dyrby CO, Koo S. A framework for the in vivo pathomechanics of osteoarthritis at the knee. Ann Biomed Eng. 2004;32:447–457. 15095819 10.1023/b:abme.0000017541.82498.37

[20] Appleyard RC, Burkhardt D, Ghosh P, Read R, Cake M, Swain MV, et al Topographical analysis of the structural, biochemical and dynamic biomechanical properties of cartilage in an ovine model of osteoarthritis. Osteoarthritis Cart. 2003;11:65–77. 10.1053/joca.2002.0867 12505489

[21] Burr DB. Anatomy and physiology of the mineralized tissues: role in the pathogenesis of osteoarthrosis. Osteoarthritis Cart. 2004;12 Suppl A:S20–30. 10.1016/j.joca.2003.09.016 14698637

[22] Hunziker EB, Quinn TM, Häuselmann HJ. Quantitative structural organization of normal adult human articular cartilage. Osteoarthritis Cart. 2002;10:564–572. 10.1053/joca.2002.0814 12127837

[23] Revell PA, Pirie C, Amir G, Rashad S, Walker F. Metabolic activity in the calcified zone of cartilage: observations on tetracycline labelled articular cartilage in human osteoarthritic hips. Rheumatol Int. 1990;10:143–147. 2124371 10.1007/BF02274838

[24] Chen R, Chen S, Chen XM, Long X. Study of the tidemark in human mandibular condylar cartilage. Arch Oral Biol. 2011;56:1390–1397. 10.1016/j.archoralbio.2011.04.007 21561599

[25] Bullough PG, Jagannath A. The morphology of the calcification front in articular cartilage. Its significance in joint function. J Bone Joint Surg Br. 1983;65:72–78. 6337169 10.1302/0301-620X.65B1.6337169

[26] O’Connor KM. Unweighting accelerates tidemark advancement in articular cartilage at the knee joint of rats. J Bone Min Res. 1997;12:580–589. 9101369 10.1359/jbmr.1997.12.4.580

[27] Meachim G, Allibone R. Topographical variation in the calcified zone of upper femoral articular cartilage. J Anat. 1984;139 (Pt 2):341–352. 6386776 PMC1164380

[28] Doube M, Firth EC, Boyde A. Variations in articular calcified cartilage by site and exercise in the 18-month-old equine distal metacarpal condyle. Osteoarthritis Cart. 2007;15:1283–1292. 10.1016/j.joca.2007.04.003 17517523

[29] Hayami T, Pickarski M, Zhuo Y, Wesolowski GA, Rodan GA, Duong le T. Characterization of articular cartilage and subchondral bone changes in the rat anterior cruciate ligament transection and meniscectomized models of osteoarthritis. Bone 2006;38:234–243. 16185945 10.1016/j.bone.2005.08.007

[30] Lane LB, Bullough PG. Age-related changes in the thickness of the calcified zone and the number of tidemarks in adult human articular cartilage. J Bone Joint Surg Br. 1980;62:372–375. 7410471 10.1302/0301-620X.62B3.7410471

[31] Martinelli MJ, Eurell J, Les CM, Fyhrie D, Bennett D. Age-related morphometry of equine calcified cartilage. Equine Vet J. 2002;34:274–278. 12108746 10.2746/042516402776186100

[32] Goldring MB, Goldring SR. Articular cartilage and subchondral bone in the pathogenesis of osteoarthritis. Ann N Y Acad Sci. 2010;1192:230–237. 10.1111/j.1749-6632.2009.05240.x 20392241

[33] Anderson HC. Matrix vesicles and calcification. Curr Rheum Rep. 2003;5:222–226. 10.1007/s11926-003-0071-z 12744815

[34] Goldring SR. Alterations in periarticular bone and cross talk between subchondral bone and articular cartilage in osteoarthritis. Ther Adv Musculoskelet Dis. 2012;4:249–258. 10.1177/1759720X12437353 22859924 PMC3403248

[35] Beckett J, Jin W, Schultz M, Chen A, Tolbert D, Moed BR, et al Excessive running induces cartilage degeneration in knee joints and alters gait of rats. J Orthop Res. 2012;30:1604–1610. 10.1002/jor.22124 22508407

[36] Han S-K, Seerattan R, Herzog W. Mechanical loading of in situ chondrocytes in lapine retropatellar cartilage after anterior cruciate ligament transection. J R Soc Interface 2010;7:895–903. 10.1098/rsif.2009.0458 19933220 PMC2871805

[37] Oettmeier R, Abendroth K, Oettmeier S. Analyses of the tidemark on human femoral heads. II. Tidemark changes in osteoarthrosis—a histological and histomorphometric study in non-decalcified preparations. Acta Morphol Hung. 1989;37:169–180. 2486460

[38] Appleton CT, McErlain DD, Pitelka V, Schwartz N, Bernier SM, Henry JL, et al Forced mobilization accelerates pathogenesis: characterization of a preclinical surgical model of osteoarthritis. Arthritis Res Ther. 2007;9:R13 17284317 10.1186/ar2120 PMC1860072

